# Interpersonal violence: an important risk factor for disease and injury in South Africa

**DOI:** 10.1186/1478-7954-8-32

**Published:** 2010-12-01

**Authors:** Rosana Norman, Michelle Schneider, Debbie Bradshaw, Rachel Jewkes, Naeemah Abrahams, Richard Matzopoulos, Theo Vos

**Affiliations:** 1 University of Queensland, School of Population Health, Herston, Queensland 4006, Australia; 2 Alcohol and Drug Research Unit, Medical Research Council of South Africa, Tygerberg, Cape Town, South Africa; 3 Burden of Disease Research Unit, Medical Research Council of South Africa, Tygerberg, Cape Town, South Africa; 4 Gender and Health Research Unit, Medical Research Council of South Africa, Tygerberg, Cape Town and Pretoria, South Africa

## Abstract

**Background:**

Burden of disease estimates for South Africa have highlighted the particularly high rates of injuries related to interpersonal violence compared with other regions of the world, but these figures tell only part of the story. In addition to direct physical injury, violence survivors are at an increased risk of a wide range of psychological and behavioral problems. This study aimed to comprehensively quantify the excess disease burden attributable to exposure to interpersonal violence as a risk factor for disease and injury in South Africa.

**Methods:**

The World Health Organization framework of interpersonal violence was adapted. Physical injury mortality and disability were categorically attributed to interpersonal violence. In addition, exposure to child sexual abuse and intimate partner violence, subcategories of interpersonal violence, were treated as risk factors for disease and injury using counterfactual estimation and comparative risk assessment methods. Adjustments were made to account for the combined exposure state of having experienced both child sexual abuse and intimate partner violence.

**Results:**

Of the 17 risk factors included in the South African Comparative Risk Assessment study, interpersonal violence was the second leading cause of healthy years of life lost, after unsafe sex, accounting for 1.7 million disability-adjusted life years (DALYs) or 10.5% of all DALYs (95% uncertainty interval: 8.5%-12.5%) in 2000. In women, intimate partner violence accounted for 50% and child sexual abuse for 32% of the total attributable DALYs.

**Conclusions:**

The implications of our findings are that estimates that include only the direct injury burden seriously underrepresent the full health impact of interpersonal violence. Violence is an important direct and indirect cause of health loss and should be recognized as a priority health problem as well as a human rights and social issue. This study highlights the difficulties in measuring the disease burden from interpersonal violence as a risk factor and the need to improve the epidemiological data on the prevalence and risks for the different forms of interpersonal violence to complete the picture. Given the extent of the burden, it is essential that innovative research be supported to identify social policy and other interventions that address both the individual and societal aspects of violence.

## Background

Decades of apartheid, political violence, and state-sponsored oppression in South Africa have contributed to a situation in which, for many people, violence is a first-line strategy for resolving conflict. In this study, we focus on exposure to interpersonal violence, which includes acts of family violence such as child abuse and intimate partner violence (IPV) as well as violence that occurs among unrelated individuals in the community [1]. Burden of disease estimates for South Africa have highlighted the particularly high rates of homicide compared with other regions of the world, with age-standardized homicide rates (64.8 per 100,000) being seven times higher than the global average [2]. While burden of disease estimates are seldom available at the country level, it is apparent that South Africa has among the highest burdens of interpersonal violence injury in the world.

Youth violence, particularly among males, is exceptionally high in South Africa, with the highest homicide rates (184 per 100,000, nine times the global rate) in males aged 15-29 years [2]. All age groups are adversely affected, and among children younger than 5 years, the homicide rates of 14.0 among boys and 11.7 per 100,000 among girls were more than double the average for low- to middle-income countries [1]. High levels of gender-based violence are also observed, notably rape, IPV, and child sexual abuse (CSA). One out of every 2 women killed in South Africa is killed by an intimate partner, resulting in the highest reported intimate femicide rate in the world: 8.8 per 100,000 women [3].

The health impact of interpersonal violence can be measured in different ways. Incidence and mortality provide a crude measure of the physical burden of interpersonal violence, while disability-adjusted life years (DALYs) incorporate both injury disability and premature mortality. Interpersonal violence injury caused about 1.0 million (6.5% of all) DALYs in South Africa in 2000 [2]. This estimate reflects the direct physical injury burden where interpersonal violence injuries are selected as the underlying cause, but it excludes the excess burden resulting from the increased risk of mortality and disability from various health outcomes associated with exposure to nonfatal violence.

A growing body of evidence indicates that violence survivors are at an increased risk of a wide range of psychological and behavioral problems, including depression, alcohol abuse, anxiety, and suicidal behavior, as well as reproductive health problems and sexually transmitted infections [1,4-12].

Studies conducted in South Africa have shown that women with violent partners are at an increased risk of HIV infection [13]. Abusive men are more likely to have HIV risk behaviors and impose risky sexual practices on partners [14-17]. Furthermore, women who have experienced violence also engage in more risky sexual practices [15]. Recent analyses by Jewkes et al. provide evidence of a significant association between CSA and risk of incident HIV infection as well as IPV and risk of incident HIV infection in young South African women [18,19].

Comparative risk assessment (CRA) methodology was developed to provide a reliable and comparable analysis of the contribution of a range of risk factors to ill health [20]. However, interpersonal violence was not among the selected major risk factors included in the World Health Organization (WHO) CRA study [20,21], although CSA was quantified [4]. There have been criticisms of previous burden of disease studies for failure to provide a comprehensive picture of burden of disease and injury among women and for omitting the contribution of IPV as a risk factor [11,22,23]. As a result, the health impact of IPV [11] and the burden attributable to both CSA and IPV [24] were estimated in Australia. Given that levels of violence in a society are modifiable and preventable, and given the high levels of interpersonal violence in South Africa, the full impact of interpersonal violence needs to be measured and recognized as a priority for effective intervention. Hence, the South African CRA included interpersonal violence as a risk factor [25,26].

In line with the Global Burden of Disease 2005 [27] approach, we continue to use categorical attribution of deaths and DALYs to the interpersonal violence injury cause but also treat exposure to nonfatal interpersonal violence as a risk factor for other diseases and injuries using counterfactual estimation and CRA methods. This paper extends initial estimates published in a letter to the editor in the South African CRA study [25]. It includes revised estimates and improved detailed methodology using recent data on the risk of HIV in women exposed to IPV and CSA. The overall aim of this study was to estimate the contribution of interpersonal violence as a risk factor to the total burden of disease in South Africa. A risk assessment of the health impact of violence has not previously been conducted in a low- or middle-income country, such as South Africa, and will therefore add to the growing international body of evidence related to the impact of violence on health.

## Methods

The World Health Organization framework of interpersonal violence was adapted [1]. Interpersonal violence refers to violence between individuals and is subdivided into family and community violence. The former includes child maltreatment, intimate partner violence, and elder abuse. Community violence includes youth violence, xenophobic violence, assault and rape by acquaintances and strangers, violence related to property crimes, and violence in workplaces and other institutions. Violent acts may be physical, sexual, emotional, or psychological in nature, or may involve deprivation or neglect. The total burden of disease and injury attributable to interpersonal violence as a risk factor was calculated using both categorical and counterfactual approaches [28].

### Categorical attribution of injury mortality and burden

Interpersonal violence itself appears as one of the mutually exclusive, categorically assigned disease and injury categories in the South African burden of disease study [2,29]. Multiple sources of information were used to derive estimates for the level and causes of injury mortality and DALYs in South Africa for the year 2000 as described elsewhere [2,29]. This injury burden was categorically attributed to "unspecified interpersonal violence" once intimate femicides (female homicides resulting from intimate partner violence) had been removed. This was done by applying the proportion of femicides perpetrated by an intimate partner from the study by Abrahams et al. [3] to the total number of femicides in South Africa in 2000. Intimate femicides were categorically attributed to IPV exposure in females.

Data limitations have restricted the scope of the study [25]. Apart from IPV where the victim is female, data on perpetrator-victim relationships were often not available, and hence, we were unable to distinguish the majority of fatal and nonfatal injuries due to other family violence from community violence. The category "unspecified interpersonal violence injuries" therefore included physical injuries and homicides related to IPV where the victim is male, as well as child and elder abuse and injuries related to community violence in males and females. Injury burden related to organized gang violence (a form of collective violence) but that could often not be differentiated from youth or community violence were also included in this "unspecified interpersonal violence" injury category.

### Counterfactual estimation

The additional health impact related to exposure to nonfatal family violence, namely CSA (contact and intercourse types in males and females) and IPV (of a physical and sexual nature, for females only) as risk factors was quantified using counterfactual estimation and comparative risk assessment methodology [20,21]. The attributable burden was estimated by comparing the current health status with a theoretical minimum counterfactual with the lowest possible risk. For both IPV and CSA, the theoretical minimum was defined by the counterfactual status of no previous or current exposure to these types of violence in the population. The population-attributable fraction (PAF) was determined by the prevalence of exposure to these risk factors in the population and the relative risks of disease occurrence given exposure.

#### Population attributable fractions

The PAFs by age and cause were calculated using customized MS Excel spreadsheets based on templates from the Australian study [24] using the formula:

$$PAF=\frac{\sum_{i=1}^{k}p_{i}(RR_{i}-1)}{\sum_{i=0}^{k}p_{i}(RR_{i}-1)+1}$$

where *p_i _*is the prevalence of exposure level i, *RR_i _*is the relative risk of disease in exposure level i, and k is the total number of exposure levels [30].

#### Prevalence of exposure

For the prevalence of CSA in males and females, we identified the Jewkes et al. 2006 study [31] as the best available data source based on study design and methodological rigor. The study recruited 15- to 26-year-old males and females into a cluster randomized and controlled trial to determine the effectiveness of a behavioral intervention, Stepping Stones, in preventing HIV infections and promoting safer sexual behavior among youth in rural Eastern Cape, South Africa. Childhood adversity was measured on a modified version of the short form of the Childhood Trauma Questionnaire [32] with specifically trained interviewers. It used a narrow definition of CSA including only two categories of abuse: contact abuse that includes touching or fondling; and intercourse that includes oral, anal, or vaginal intercourse. Noncontact abuse that encompasses a range of acts and includes inappropriate sexual solicitation or indecent exposure was excluded.

We assumed no trend in the prevalence of CSA over time, so the same estimates of prevalence from the Stepping Stones study were used across adult age groups 15 years and older. In the absence of data on prevalence of exposure in children under 5 years, CSA prevalence was assumed to be zero below the age of 5 years (Table 1).

**Table 1:** Estimated prevalence and 95% confidence intervals by CSA type, age, and sex, South Africa, 2000

Male		Age (in years)	
CSA type	**0-4**^**b**^	5-14	15+
Not exposed	-	95.1%	88.0%
Contact	-	3.1 (2.0-4.2%)	7.8 (6.0-9.5%)
Intercourse	-	1.8 (0.9-2.6%)	4.3 (3.0-5.6%)

**Female**^**a**^		**Age (in years)**	
**CSA type**	**0-4**^**b**^	**5-14**	**15+**

Not exposed	-	88.8%	66.4%
Contact	-	5.2 (3.9-6.4%)	15.5 (13.5-17.5%)
Intercourse	-	6.0 (4.7-7.4%)	18.1 (15.9-20.3%)

Source: Unpublished data from Stepping Stones study (Rachel Jewkes, personal communication, 2006)

^a ^In prevalence estimates of CSA in females, there may be overlap with exposure to IPV

^b ^Estimates not available for this age group

For the prevalence of exposure to IPV in females, we used two data sources yielding low and high estimates for subsequent use in a sensitivity analysis. For the low estimates, we re-analyzed data from the three-province survey on IPV [33] conducted among rural women 18-49 years of age in Eastern Cape, Mpumalanga, and the Northern Province. We used two categories of exposure to IPV, namely current exposure to physical and/or sexual violence by an intimate partner (in the last 12 months) and previous exposure (more than 12 months ago) to physical and/or sexual violence by an intimate partner. Intimate partners included current or ex-partners. In order to match age groups required for the attributable burden assessment, females 15-17 years of age were assumed to have the same prevalence as women 18-29 years of age. Similarly, women aged 50-59 years were assumed to have the same prevalence as 45-49-year-old women in the survey. In the absence of data on the prevalence of IPV for women older than 49 years, we followed a conservative approach and assumed no current exposure to IPV in women 60 years and older (Table 2). This is supported by data that have recently become available from a study in Gauteng Province where women over age 55 report no past year exposure to IPV (Rachel Jewkes, personal communication, 2010).

**Table 2:** Estimated prevalencea (%) of IPV among women by age, South Africa, 2000

	In past 12 months			More than 12 months ago		
Age (in years)	Mean	Low	High	Mean	Low	High
15-29	18.4	9.4	27.3	21.2	15.6	26.8
30-44	22.3	11.5	33.1	17.2	12.6	21.8
45-59	21.4	11.0	31.8	22.5	16.5	28.5
60+	0.0	0.0	0.0	40.1	24.6	55.5
**15+**	**18.4**	**9.5**	**27.4**	**21.9**	**15.6**	**28.1**

Source for low estimate: Jewkes et al. [33]

Source for high estimate: Dunkle et al. [34]

^a^Estimated IPV prevalence may also include exposure to CSA

For the high estimate, we used data from a study carried out among pregnant women attending antenatal clinics in the urban area of Soweto [34] that included questions similar to the WHO violence against women instrument [35]. Only overall prevalence of current (30.1%) and previous (25.4%) IPV for pregnant women over 16 years of age was available. We imposed the age pattern from the three-province study to derive age-specific estimates (Table 2).

Girls who experience CSA are more likely to experience IPV as adults compared with nonabused girls [22]. Several studies also suggest that women who experience multiple types of abuse are at a higher risk of depressive symptoms and other mental disorders compared to women who only experience one type of abuse [36]. Following the method used in the Australian burden of disease study [24], in order to avoid overestimating the burden attributable to these two forms of violence, we determined the prevalence of single exposure to IPV and CSA as well as dual exposure. In the study of women in antenatal clinics in Soweto [34], the risk ratio of violent revictimization through IPV for women who experienced CSA (contact and intercourse types) was 2.43 (95% CI: 1.93-3.06). We applied this risk ratio to estimate the prevalence of dual exposure (Table 3). Assuming no trend over time in the prevalence of CSA, the prevalence of dual exposure was subtracted from the CSA and IPV prevalence estimates presented in Table 1 and 2 to derive single exposures.

**Table 3:** Estimated prevalence (%) of IPV and CSA among women by age, South Africa, 2000 (women who have experienced both types of abuse)

Age (in years)	IPV in past 12 months and CSA	IPV more than 12 months ago and CSA	IPV and CSA ever
15-29	10.1	11.7	21.8
30-44	12.3	9.5	21.8
45-59	11.8	12.4	24.2
60+	0.0	22.1	22.1
**15+**	**9.8**	**12.4**	**22.2**

#### Relative risk estimates

For contact and intercourse CSA, we made use of relative risks (RRs) (adjusted for family dysfunction and other types of abuse) published in the global assessment [4] based on a systematic review and meta-analysis of published studies. The same adjusted RRs were used for males and females and across age groups.

In the absence of local studies estimating the magnitude of the association of IPV and major health outcomes, we relied on the analysis of the Australian Longitudinal Study on Women's Health. This was a multinomial logistic regression analysis to compute the relative risk of health outcomes comparing women reporting exposure to previous or current IPV with those reporting no such exposure to violence after controlling for socioeconomic variables (level of education, employment status, occupation, marital status, language spoken, indigenous status, place of residence) as well as smoking and drinking status [11]. The RRs were assumed to apply to women of all ages except for tobacco smoking, for which an exponential decline was assumed with age [11].

For certain mental health outcomes, the published RRs related to exposure to CSA [4] and IPV [11] in females were adjusted to derive RR estimates for the combined exposure state of having experienced both CSA and IPV, following the method used in the Australian study [24]. Briefly, the mean psychological function indices and standard errors reported by Messman-Moore and colleagues for women who had experienced both child and adult abuse [36] were used to calculate Hedges' adjusted g for the standardized mean difference (a combined effect size) [37]. These effect sizes were then converted into odds ratios [38] for the risk of depression, anxiety, and post-traumatic stress disorder (PTSD) by exposure group. The calculated odds ratios, along with the published relative risks for CSA [4] and IPV [11] were then used to derive RR estimates for CSA only, IPV only, and CSA and IPV combined. The relative risks for alcohol use disorders, other drug use disorders, and self-inflicted injuries were extrapolated from the depression relative risks for CSA only, IPV only, and combined CSA and IPV. For ease of reporting, the PAF calculated for the combined CSA and IPV category was proportionately redistributed to either CSA or IPV.

In order to calculate PAFs for the physical injury disability related to IPV, we used the prevalence of current IPV presented in Table 2 and the average of the RRs reported for having sustained bruises, lacerations, and fractures in an Australian emergency department (RR = 2.50, 95% CI: 1.03-6.26) based on the proportion of hospitalizations for assaults where the victim-perpetrator relationship was recorded as spouse or intimate partner [39] as used by Vos and colleagues [11].

A recent longitudinal analysis of data from 1,099 young South African women from the Stepping Stones trial who were HIV negative at baseline and had subsequent HIV test results showed that IPV increased the risk of incident HIV infection (incidence rate ratio 1.51, 95% CI: 1.04-2.21) after adjusting for herpes simplex virus type 2 (HSV2) infection at baseline, age, treatment, stratum, and person years of exposure (Table 4), allowing the inclusion of this important outcome in our analysis[18]. Since HIV spread rapidly between 1990 and 2000 in South Africa, a conservative approach has been adopted by applying the risk of HIV to current exposure to IPV and not previous exposure.

**Table 4:** Relative risk estimates for the association between IPV and health outcomes

Condition	IPV in last 12 months RR (95% CI)	IPV more than 12 months ago RR (95% CI)
Tobacco smoking	2.98 (2.09-4.25)	2.79 (2.33-3.34)
Alcohol abuse	1.82 (1.04-3.18)	1.47 (1.03-2.10)
Illicit drug use	2.27 (1.63-3.17)	1.23 (1.02-1.48)
Depression	3.05 (2.18-4.28)	1.96 (1.59-2.42)
Anxiety disorders	2.59 (1.59-4.20)	1.83 (1.36-2.47)
Eating disorders^a^	1.87 (1.39-2.51)	1.22 (1.04-1.43)
Sexually transmitted infections	2.24 (1.40-3.58)	1.54 (1.15-2.08)
Abnormal Pap smear*	1.43 (1.03-2.00)	1.46 (1.22-1.75)
Deliberate self-harm	7.05 (4.55-10.93)	2.53 (1.81-3.56)
HIV/AIDS^b^	1.51 (1.04-2.21)	-

Source: Adapted from Vos et al. [11]

*Used as a proxy for cervical cancer [11]

^a ^Not applied in this study due to lack of burden estimates

^b ^Estimate from Jewkes et al. [18] adjusted for herpes simplex virus type 2 infection at baseline (an important STI), age, treatment, stratum, and person years of exposure.

The incidence of HIV was also found to be significantly higher in women who experienced CSA (incidence rate ratio 1.66, 95% CI: 1.04-2.63) after controlling for age, education, parental death, and socioeconomic status, but there was not sufficient evidence of a causal relationship with CSA in males, so this outcome was only included for females [19].

#### Attributable burden

The PAFs were applied to estimates of the burden of disease in South Africa for the selected health outcomes, measured in deaths, years of life lost (YLL), years lived with disability (YLD), and DALYs [29]. In the case of tobacco smoking and alcohol use associated with IPV and alcohol use associated with CSA, the PAFs were applied to the burden attributable to tobacco and alcohol, respectively, calculated in the South African CRA study [26].

#### Uncertainty Analysis

Monte Carlo simulation-modeling techniques were used to present uncertainty ranges around point estimates reflecting the main sources of uncertainty in the calculations. Ersatz software version 1.0 [40] was used as an add-in to Excel, allowing multiple recalculations of the Excel spreadsheet, each time choosing a randomly drawn value from the distributions defined for input variables. A uniform probability distribution was specified between the low and high IPV prevalence estimates. For the CSA and IPV exposure categories, a Dirichlet distribution (a conjugate of the multinomial distribution) was specified that ensures that the returned random deviates (with binomial distributions) always sum to 1 [40]. We assumed that the homicide and interpersonal violence injury estimates in 2000 [2] could vary by an arbitrary 10% and specified a triangular distribution with three points (minimum, most likely, and maximum). For the relative risk input variables, we made the standard assumption that the natural logarithm of the RR has a normal distribution and used standard errors derived from the published 95% CIs. We used the Ersatz random function ErRelativeRisk with a correction that takes the RR and SE [ln(RR)] as parameters and recalculates them to produce a mean effect size equal to the point estimate of the RR in the uncertainty analysis [41]. For each of the output variables (namely attributable burden as a percentage of total burden in South Africa in 2000), 95% uncertainty intervals were calculated bounded by the 2.5^th ^and 97.5^th ^percentiles of 2,000 iteration values generated.

## Results

Interpersonal violence (including not only the injury burden but also some of the long-term mental and behavioral consequences) was an important risk to health in South Africa and accounted for an estimated 870,000 DALYs or 10.2% (95% uncertainty interval: 9.8%-10.7%) of all DALYs in males and about 840,000 or 10.9% (95% uncertainty interval: 6.8%-14.9%) of all DALY in females, in 2000 (Table 5). IPV and CSA have a significant impact on the health of South African women. For women of all ages, CSA accounted for 3.5% and IPV for 5.4% of the total disease and injury burden.

**Table 5:** Estimated burden attributable to interpersonal violence by sex, South Africa, 2000

	Male			Female		
	**PAF**^**c**^	Deaths	**DALYs**^**d**^	**PAF**^**c**^	Deaths	**DALYs**^**d**^
**Intimate partner violence**		**0**	**0**		**10 187**	**418 575**

Major depression	-	-	-	21.0%	0	42 073
Anxiety disorders^b^	-	-	-	14.3%	0	12 133
Alcohol consumption	-	-	-	9.8%	658	17 694
Drug use disorders	-	-	-	13.7%	0	3 094
Self-inflicted injuries	-	-	-	36.2%	448	11 105
Tobacco smoking	-	-	-	22.7%	1 747	34 458
Cervical cancer	-	-	-	15.7%	551	8 460
HIV/AIDS	-	-	-	7.2%	4 897	197 080
Sexually transmitted infections	-	-	-	19.8%	42	6 194
Femicides	-	-	-	45.3%	1 845	51 833
Physical injury disability	-	-	-	16.5%	0	34 450

**Child sexual abuse**		**1 279**	**47 327**		**6 112**	**272 348**

Major depression	5.7%	0	6 713	8.9%	0	17 935
Alcohol consumption	5.6%	979	25 161	6.8%	463	12 171
Drug use disorders	7.8%	1	4 954	11.0%	0	2 472
Post-traumatic stress disorder	22.4%	0	1 354	23.8%	0	4 210
Panic disorder	10.2%	0	1 751	14.4%	0	5 154
Self-inflicted injuries	7.0%	299	7 394	13.5%	168	4 145
HIV/AIDS	-	-	-	8.3%	5 480	226 260

**Unspecified interpersonal violence^a^**		**23 041**	**819 141**		**2 676**	**150 288**

Interpersonal violence injuries	100%	23041	819 141	100%	2676	150 288

**Total**		**24 320**	**866 468**		**18 975**	**841 210**
95% uncertainty interval		23 000-26 000	828 000-903 000		11 000-27 000	527 000-1 152 000

**% of total burden**		**8.9%**	**10.2%**		**7.7%**	**10.9%**
95% uncertainty interval		8.4%-9.3%	9.8%-10.7%		4.6-10.8%	6.8-14.9%

^a^Unspecified interpersonal violence = unspecified community and family violence (injury burden) where victim-perpetrator relationship is unknown

^b^Anxiety disorders = panic disorders + obsessive compulsive disorder + post-traumatic stress disorder

^c^PAF = Population attributable fraction; **^d^**DALYs =disability-adjusted life years

For IPV, the highest PAF was for femicides (45%), followed by self-inflicted injuries (36%) and tobacco smoking (23%). Exposure to physical and sexual IPV accounts for a substantial proportion of the HIV/AIDs burden (7%). For CSA, the highest PAF was for PTSD (22% and 24% in males and females, respectively) followed by panic disorders (10% and 14% in males and females, respectively). An estimated 6% of all major depression burden in males and 9% in females could be attributed to CSA. In females, about 8% of all HIV/AIDs burden was attributed to CSA.

**Figure 1:**
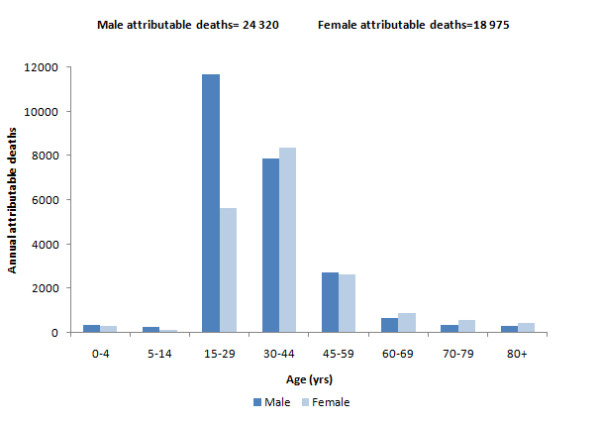
**Interpersonal violence-attributable deaths by age and sex, South Africa, 2000**.

The number of deaths and DALYs attributable to interpersonal violence is a function of both the attributable fractions and the amount of burden of disease accounted for by the related health outcomes. Attributable mortality peaked in young males aged 15-29 years, reflecting the high injury burden from youth violence, while in females, the peak was in the 30-44-year age group. With the addition of HIV/AIDs as an outcome related to exposure to CSA and IPV in females, attributable mortality in females is slightly higher than in males 30 years and older (Figure 1).

IPV accounted for 50% and CSA for 32% of the total interpersonal violence-attributable burden in females, highlighting the large problem of gender-based violence against women. In males, 95% of the total attributable burden was from "unspecified interpersonal violence" injuries.

**Figure 2:**
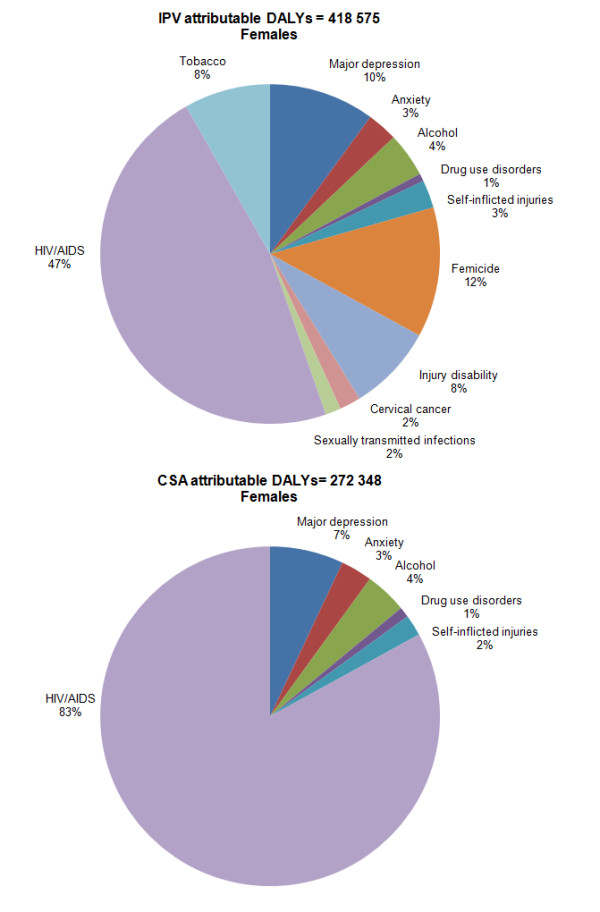
**Burden attributable to IPV and CSA in females, South Africa, 2000**.

More than half the burden attributable to CSA in males was alcohol-related (53%), followed by self-inflicted injuries (16%) and major depression (14%). HIV/AIDS accounted for the largest proportion of the burden attributable to CSA in females (83%), followed by major depression (7%) and alcohol (4%). HIV/AIDS and sexually transmitted infections, including cervical cancer, accounted for more than half (51%) of the burden attributable to IPV in females. Poor mental health was also an important contributor to the total burden, with major depression, anxiety, and self-inflicted injuries together contributing 16% of the total disease burden associated with IPV. Substance abuse, including alcohol, drugs, and tobacco consumption, accounted for 13% of the burden (Figure 2).

## Discussion

While CRA methodology has previously been applied to selected forms of family violence [4,11,20], this study breaks new ground in its attempt to measure the full health impact of interpersonal violence. It combines the additional burden from the long-term health consequences and the direct physical injury burden to present a more comprehensive estimate of the contribution of interpersonal violence to the burden of disease in South Africa.

The study shows that in the year 2000, an estimated 43,000 deaths, or 8.3% (95% uncertainty interval: 6.8%-9.8%) of all deaths in South Africa in 2000, were attributed to interpersonal violence as a risk factor. Of the 17 risk factors included in the South African CRA study, interpersonal violence was the second leading cause of healthy years of life lost, after unsafe sex, accounting for an estimated 1.7 million DALYs or 10.5% of all DALYs (95% uncertainty interval: 8.5%-12.5%) in 2000 when excess mortality and disability from other causes was taken into account.

The revised estimate of 1.7 million DALYs is higher than the overall figure of 1.4 million (8.4% of all) DALYs initially attributed to violence in the South African CRA study [25]. This is due to methodological improvements and the availability of more recent data as detailed in the methods section of this paper.

International comparisons for interpersonal violence as a risk factor are not possible, but comparisons can be made for the CSA and IPV subtypes. For CSA, PAFs were slightly lower than those reported by Andrews et al. for the African region due to the higher prevalence estimate of CSA used by Andrews et al., although the authors state that data for this subregion came from a few studies that were poor methodologically [4]. As reported by Andrews et al., CSA also contributes to a higher percentage of DALYs for females (3.5%) than for males (0.6%), driven by the higher prevalence of CSA in females and the inclusion of HIV/AIDs as an outcome in females in this study. For IPV, PAFs were similar to those reported by Vos et al. [11]. For women of all ages, IPV accounted for 5.4% of the total disease and injury burden in South Africa compared to 2.9% in the Australian study [11]. This is due to the higher prevalence of IPV in this study and the inclusion of HIV/AIDs as a related health outcome. The very high burden of HIV/AIDs attributed to interpersonal violence among women in this study reflects the context of high HIV prevalence in South Africa.

The burden attributable to interpersonal violence as a risk factor in this study is an underestimate. It is difficult to quantify the full impact of nonfatal violence as many acts of violence are not reported, and data are incomplete. In particular, it has not been possible to quantify the longer-term health consequences of exposure to the following types of violence: youth and community physical violence, disproportionately affecting men; IPV in male victims; sexual violence by acquaintances and strangers in adult women and any sexual violence in adult men; and other forms of child maltreatment and elder abuse. For these forms of violence, only the direct injury consequences are included in our estimates. Exposure to nonfatal violence of a psychological or emotional nature or involving deprivation or neglect for any type of interpersonal violence could not be quantified due to lack of data on prevalence of exposure and hazard size.

Exposure to community violence among males has been associated with mental health and other health outcomes [42-45]. A recent study in South Africa has demonstrated associations of PTSD with political detention and torture among males [46]. This study also found that frequent exposures to criminal assault and childhood abuse were associated with the greatest number of PTSD cases among men at a population level (ibid).

In addition to CSA, other forms of child maltreatment (namely physical and emotional abuse, deprivation, and neglect) are also risks to health [9] but were not quantified in this study. In the Jewkes et al. 2010 analysis of Stepping Stones data, emotional neglect in childhood was associated with depression, suicidality, alcohol abuse, and incident HSV2 infections in women and depression and drug use in men. Incident HIV infections were more common in women who experienced not only sexual abuse but also emotional and physical punishment in childhood [19].

The new Global Burden of Disease 2005 study [27] is undertaking systematic reviews of the risks associated with exposure to IPV and sexual violence, an update of the CSA systematic review, and scoping studies to determine the strength of the evidence of a causal relationship between exposure to other forms of child maltreatment, youth and community violence, and various health outcomes. This work will improve quantification of these health risks in future studies.

Although attempts have been made to quantify sampling uncertainty in this study, there is clearly some uncertainty around these estimates beyond sampling uncertainty that could not be quantified. Extrapolating overall risks from other countries to South Africa in the absence of reliable local estimates of the risk of exposure to this risk factor is an important source of uncertainty. There is also some uncertainty around cause of death and burden of disease estimates [29,47], although uncertainty around the interpersonal violence injury estimates for 2000 was included in this analysis. In general, however, the study could be improved through more representative data on exposure to violence and the epidemiological relationship between the risk factor and health. These and other study limitations are discussed in more detail below.

In the absence of South African data on various health impacts of CSA, RR estimates from the systematic review and meta-analysis of Andrews et al. [4] were used to increase international comparability. A systematic review of risks associated with exposure to IPV has not yet been carried out, and in the absence of local data, we used risks from the Australian study of Vos et al. [11], although differences in risk are likely to exist across subpopulations. Particularly for IPV, risks among exposed South African women may be different than risks among Australian women. Recently, data have become available from an analysis of Stepping Stones data by Jewkes et al. in 2010 showing that CSA is associated with alcohol abuse in men and depression and alcohol abuse in women [19]. The magnitude of the risk of depression was comparable to that reported by Andrews and colleagues. But the risk of alcohol abuse in both men and women was much greater (adjusted OR 3.68 [95% CI: 2.00-6.77] in men and OR 3.94 [95% CI: 1.90-8.17] in women) in the Jewkes et al. study among individuals exposed to frequent CSA compared with 1.87 (95%CI: 1.47-2.39) for intercourse type of CSA in the Andrews et al. meta-analysis. It should be noted that the Jewkes et al. study uses a slightly broader definition of CSA that included sex with a partner five or more years older and also asks participants how often they had experienced abuse. Regarding the risk of HIV infection, although this cause was not included by Andrews et al., the Jewkes et al. findings are similar to those of Reza et al. in Swaziland [48] and studies from the US [49-53].

Another important limitation is that the majority of studies examining the relationship between exposure to family violence and disease outcomes have been cross-sectional analyses that by definition cannot prove a temporal relationship between exposure to violence and the onset of health outcomes. However, exposure to CSA occurs during childhood and usually prior to the onset of any adult psychiatric disorder. Thus, in this instance, cross-sectional studies may be of use, although some psychiatric disorders may have had an onset date preceding the violence. For IPV, where temporality becomes a greater concern, the consistency of the findings across multiple disease outcomes in the study by Vos and colleagues and the observed gradient of risk for mental health outcomes with more severe, more recent, or ongoing exposure to IPV provide support for a causal relationship. The Jewkes et al. 2010 longitudinal analysis also provides strong temporal evidence to support a causal relationship between IPV and incident HIV infection [18,19]. The relation between the variables was plausible and coherent, and research from several settings has shown consistency and supports the strength of association [18].

Exposure to these forms of family violence often co-occurs within the context of other family dysfunction, social deprivation, and other environmental stressors also associated with mental disorders [54]. It remains possible that some of the effect of CSA and IPV on adult health may still be explained by confounding despite attempts to control for these co-occurring factors in these studies [4,11,18,19].

Accurately measuring the exposure to violence is extremely challenging, and prevalence estimates are sensitive to methodological factors that influence the reporting of abuse [35,55,56]. Ethical issues and study design have also been identified as major factors that influence disclosure of violence. Underreporting of sensitive behaviors in large surveys is reflected in the South African Demographic and Health Survey of 1998, where only 1.6% of women reported having been raped before the age of 15 years in this nationally representative sample [57], compared to 5.0% in the Dunkle et al. study of revictimization among women attending antenatal clinics in Soweto [34] that used an identical question. The overall prevalence of CSA in the Dunkle study (8.0%) was higher than population-based data would suggest but considerably lower than estimates from the Jewkes et al. 2006 study [31] used in this analysis (33.6%). We used the Jewkes et al. 2006 study of Stepping Stones data because the definition of exposure best matched the contact and intercourse levels of exposure in the Andrews et al. [4] study from which the RR estimates were derived. Higher CSA prevalence estimates have been reported in other South African research that used much broader definitions. A study in the Northern Province included unwanted kissing and found that 54.2% of 414 school students reported unwanted sexual contact, although only 13.3% of these considered themselves to have been abused [58,59]. In the more recent analysis of Stepping Stones data by Jewkes et al., sexual abuse was reported by 39% of women, using a slightly broader definition of sexual abuse that included sex with a partner five or more years older as mentioned previously [19].

The IPV prevalence estimate based on pregnant women attending antenatal clinics in Soweto [34] is likely to be somewhat higher than a national average because of the association between IPV and pregnancy. It was not implausibly high, given that more than 40% of women aged 15-26 years reported IPV in the Stepping Stones study that was conducted in 70 villages in rural South Africa [31], but it was still considerably higher than that in the study across the three provinces (Eastern Cape, Mpumalanga, and the Northern Province) [33], where the use of more general questions may have resulted in underreporting. In a sensitivity analysis considering high and low exposure scenarios, IPV-attributable burden in females varied between 250,000 and 580,000 DALYs - indicating a considerable health burden even in the lower prevalence scenario.

The roots of the high levels of crime and violence in South Africa undoubtedly lie in its colonial and apartheid past and the devastating impact of racial discrimination, impoverishment, forced removals, and the migrant labor system on families and the socialization of young men [60]. As a consequence, many young men refashioned manhood to draw on resources that were available, which increasingly meant the application of strength, courage, and male camaraderie to the criminal pursuits of gangs [61]. In a context imbued with a pervasive sense of powerlessness, many men directed their power into controlling and disciplining women through rape and domestic violence [62]. Efforts to support men in building nonviolent identities have been limited, although intervention studies show that this can be done [63]. Since 1994, there has been a substantial decline in almost all forms of violent crime except rape [64], but levels of violence remain very high. Marked income inequality and high levels of unemployment have been identified as contributory factors in the high levels of violence in the country [65] since evidence shows that as inequalities increase, the quality of social relations deteriorates and violence increases [66,67]. Drugs and alcohol and the social acceptance of most forms of violence are also major contributors. Although gender-based violence has been acknowledged as a health and human rights concern, and gender equality has been enshrined in the South African constitution, this has yet to consistently impact the experiences of South African women [68,69].

Among women, IPV and CSA account for 82% of the burden attributable to interpersonal violence with HIV/AIDs a major contributor. This demonstrates the importance of interventions to protect children and of effectively addressing the HIV epidemic through programs and interventions that address violence and gender inequity in relationships.

## Conclusions

When the contribution of IPV and CSA to burden of disease and injury is taken into account, this study indicates that a focus on only the direct physical injuries would underrepresent the burden of violence. There is value in estimating the additional burden from other related health consequences. Our findings confirm that interpersonal violence is an important public health problem in South Africa. Research is needed to understand its broader social context and to develop interventions for primary prevention and prevention of its long-term health consequences. In light of various areas of uncertainty and omissions, it remains important to improve the South African epidemiological database in subsequent CRA studies. This study highlights the enormous difficulties in measuring interpersonal violence and the need to improve the epidemiological data on the prevalence and risks for the different forms of interpersonal violence and the need for research to quantify the noninjury health consequences of youth and community violence so that the full health impact of interpersonal violence can be more comprehensively assessed. Quantifying the burden is only a first step. In terms of the public health approach to violence, it is necessary to quantify the problem, examine the causal pathways involved, and look to interventions [1]. Despite the challenges of intervention research that result from the complex linkages of underlying determinants and causes of violence and aggressive behavior [70], given the extent of the burden, it is essential that innovative research be supported to identify social policy and other interventions that address both individual and societal aspects of violence.

## List of Abbreviations

AIDS: Acquired immune deficiency syndrome; CRA: Comparative risk assessment; CSA: Child sexual abuse; DALY: Disability-adjusted life year; HIV: Human immunodeficiency virus; IPV: Intimate partner violence; PAF: Population attributable fractions; PTSD: Post-traumatic stress disorder; RR: Relative risk; WHO: World Health Organization; YLD: Years lived with disability; YLL: Years of life lost due to premature mortality.

## Competing interests

The authors declare that they have no competing interests.

## Authors' contributions

RN conceived, designed, and coordinated the study, performed the statistical analysis, and drafted the manuscript. MS made substantial contributions to the conception and design of the study and helped to draft sections of the manuscript. DB made substantial contributions to conception and design, analysis, and interpretation of data and revised the manuscript critically for important intellectual content. RJ made substantial contributions to conception and design, acquisition and interpretation of data, and revised the manuscript critically for important intellectual content. NA and RM made contributions to acquisition and interpretation of data and revised the manuscript critically for important intellectual content. TV made substantial contributions to analysis and interpretation of data and revised the manuscript critically for important intellectual content. All authors read and approved the final manuscript.
